# Investigating variability in morphological processing with Bayesian distributional models

**DOI:** 10.3758/s13423-022-02109-w

**Published:** 2022-06-17

**Authors:** Laura Anna Ciaccio, João Veríssimo

**Affiliations:** 1 Potsdam Research Institute for Multilingualism, University of Potsdam, Potsdam, Germany; 2 Present Address: Fachbereich Philosophie und Geisteswissenschaften, Brain Language Laboratory, Freie Universität Berlin, Berlin, Germany; 3 Department of Linguistics, University of Potsdam, Potsdam, Germany; 4 Center of Linguistics, School of Arts and Humanities, University of Lisbon, Lisbon, Portugal

**Keywords:** RT distribution, Distributional models, Masked priming, Visual word recognition, Morphological processing

## Abstract

We investigated the processing of morphologically complex words adopting an approach that goes beyond estimating average effects and allows testing predictions about variability in performance. We tested masked morphological priming effects with English derived (‘printer’) and inflected (‘printed’) forms priming their stems (‘print’) in non-native speakers, a population that is characterized by large variability. We modeled reaction times with a shifted-lognormal distribution using Bayesian distributional models, which allow assessing effects of experimental manipulations on both the mean of the response distribution (‘mu’) and its standard deviation (‘sigma’). Our results show similar effects on mean response times for inflected and derived primes, but a difference between the two on the sigma of the distribution, with inflectional priming increasing response time variability to a significantly larger extent than derivational priming. This is in line with previous research on non-native processing, which shows more variable results across studies for the processing of inflected forms than for derived forms. More generally, our study shows that treating variability in performance as a direct object of investigation can crucially inform models of language processing, by disentangling effects which would otherwise be indistinguishable. We therefore emphasize the importance of looking beyond average performance and testing predictions on other parameters of the distribution rather than just its central tendency.

**Supplementary Information:**

The online version contains supplementary material available at 10.3758/s13423-022-02109-w.

## Introduction

Psycholinguistic research, and cognitive science more generally, is primarily concerned with estimating average effects for population samples in a given task. The underlying assumption is that human performance is largely homogeneous—across participants, items, or trials—and, consequently, average effects can be taken to reflect the cognitive mechanisms involved in performing the task. Hence, variability in performance has not traditionally been seen as a potential source of information about the structure of the cognitive system. Instead, it has been mostly ignored or dismissed as noise (Andrews, 2012). However, recent research has witnessed increasing interest in variability in language processing, with a growing consensus that this is a reflection of a flexible system, and therefore an intrinsic aspect of language processing mechanisms (see Amenta & Crepaldi, 2016; Kidd, Donnelly, & Christiansen, 2018). This makes investigating variability crucially informative for building theoretical models of language processing.

The present work focuses on variability in morphological processing, that is, in processing complex words such as *player* [play][-er] or *played* [play][-ed], during visual word recognition. A widely employed technique in morphological processing research is the *masked priming* paradigm. In a masked priming experiment, participants are typically required to perform a lexical decision on a series of visually presented target words. These are preceded by so-called prime words, which are presented very briefly (around 50 ms) and are in turn preceded by a visual mask, thus preventing their conscious recognition (Baayen, 2014; Kinoshita & Lupker, 2004). Prime words can be morphologically related to the corresponding target, such as in *walked-WALK*, or completely unrelated in form or meaning, e.g., *kissed-WALK* (e.g., Feldman & Soltano, 1999; Rastle, Davis, Marslen-Wilson, & Tyler, 2000). When prime-target pairs are morphologically related, reaction times (RTs) are on average faster as compared to the unrelated condition, an effect known as ‘morphological priming’ (Amenta & Crepaldi, 2012). Morphological priming has been attributed to pre-activation of the target word during the processing of the morphologically related prime, due to decomposition into its morphological constituents—e.g., ‘walked’ $\rightarrow$‘walk’ + ‘ed’ (Rastle, Davis, & New, 2004; Taft & Forster, 1975)—or, according to more recent accounts, to extraction of edge-aligned embedded words from letter strings—e.g., extracting ‘walk’ from ‘walked’ (Grainger & Beyersmann, 2017). Alternatively, morphological priming effects have been explained in terms of shared semantic and orthographic properties of prime and target, without positing an independent level of morphological representation (Baayen, Milin, Urević, Hendrix, & Marelli, 2011; Baayen & Smolka, 2020; Feldman, 2000).

Masked priming effects have been reported for a series of different morphological phenomena from many typologically different languages (see Ciaccio, Kgolo, & Clahsen, 2020, for a review). All of these studies have focused on the effect of prime presentation on *average* RTs, neglecting the fact that the same average RT may come from different RT distributions, possibly with different levels of variability (standard deviation) around the mean. In recent psycholinguistic studies from other domains than masked morphological priming, the standard deviation around average effects has been shown to systematically vary as a function of experimental manipulations (Chuang, Fon, Papakyritsis, & Baayen, 2021b; Tomaschek et al., 2020). This would raise the question as to whether different types of morphological phenomena, despite showing comparable priming effects on average RTs, are associated with different levels of variability around such average effects, and if so, why.

Besides providing a more thorough picture of the underlying data, investigating variability in morphological processing is also of theoretical interest. In lexical decision experiments, mostly involving non-native (L2) speakers, RTs have been shown to become less variable on a trial-by-trial basis when lexical representations become better established, for example with practice, and thus processing becomes more efficient (Segalowitz, & Segalowitz, 1993; Segalowitz, Segalowitz, & Wood, 1998). Moreover, in novel word-learning studies, trial-level variability has been shown to increase during the process of establishing new representations (Solovyeva & DeKeyser, 2018). Variability in lexical processing has been therefore taken as a crucial index of processing automaticity, with a higher level of variability reflecting less efficient, or less automatic, lexical processing (Segalowitz, 2008). While several studies have looked at variability in performance in the context of second or artificial language learning, including some testing morphosyntax (e.g., Rodgers, 2011; for a review, see Segalowit, 2008), none of them have specifically looked at online morphological processing.

By extending the account of variability in lexical processing proposed in Segalowitz and Segalowitz (1993) and subsequent studies to the domain of morphological processing, we may take the amount of variability in RTs in a masked morphological priming paradigm to reflect how efficiently, or automatically, speakers are able to activate the morphological form presented in the prime (e.g., ‘printer’ or ‘printed’) and, from that form, activate the stem (‘print’). Note that a ‘standard’ masked priming effect on average RTs can only go as far as providing some evidence for lexical activation of a stem from the morphologically complex prime, while it does not tell us anything about how efficiently this process operates on a trial-by-trial basis. This way, analyses of *variability* around *average* RTs would crucially complement standard RT analyses of masked priming data.

A population that lends itself particularly well to the investigation of trial-level variability in language processing are L2 speakers. Performance in an L2 is characterized by larger heterogeneity than in native language, both within and across individuals (Bialystok & Hakuta, 1999; Hopp, 2013; White, 2003). When it comes to morphological processing, results from L2 speakers tend to be more variable across studies than results from native speakers. However, there is at least some indication that variability may also be selective, or at least more enhanced, for some types of morphologically complex words as opposed to others. While masked morphological priming studies with L2 speakers have generally reported robust priming effects from derived forms (Ciaccio & Clahsen, 2020; Diependaele, Duñabeitia, Morris, & Keuleers, 2011; Heyer & Clahsen, 2015; Li, Jiang, & Gor, 2017), the size of priming effects with inflected words tends to be smaller or to substantially vary across studies (Jacob, Heyer, & Veríssimo, 2018; Veríssimo, Heyer, Jacob, & Clahsen, 2018; Feldman, Kostić, Basnight-Brown, Durdević, & Pastizzo, 2010). This is in line with previous work suggesting persistent vulnerabilities in L2 speakers for inflectional morphology, especially with respect to the ability of consistently and reliably accessing inflected forms and morphosyntax (Blom, Polisšenská, & Weerman, 2006; Hopp, 2013; White, 2003). Following the approach to variability by Segalowitz and Segalowitz (1993), the inability of consistently accessing inflected forms should not only impact the size of an average experimental effect of an L2 group, but it should also be reflected in more trial-by-trial performance variability. We therefore took the inflection-derivation dichotomy in an L2 as a particularly good test case for investigating RT trial-level variability in morphological processing, and whether this varies depending on the type of complex words being processed.

## The present study

The experiment reported below contrasted the processing of English inflected and derived forms using the masked priming paradigm. We investigated the effect of these two types of morphologically complex words on the visual recognition of their bases. Specifically, we assessed priming effects elicited by inflected *-ed* past-tense forms (e.g., *printed*) and derived *-er* nominalizations (e.g., *printer*) on lexical decision times to the same target stems (e.g., *print*). The two conditions were created as to be comparable in a range of morphological and non-morphological properties: (a) both *-ed* and *-er* are regular, productive, and semantically transparent suffixes; (b) the two types of morphological primes were equivalent in their amount of orthographic overlap with their targets; and (c) priming conditions were well matched in other lexical measures (see below). Crucially, priming effects were estimated not only on average response speed but also on the variability of responses.

We made use of Bayesian *distributional models* to assess the effects of morphologically related primes. In distributional models, estimates refer not only to the mean of responses in different experimental conditions but can also describe additional features of the response distribution, for example, its variability. Moreover, these models allow the different parameters of a distribution to depend on a set of explanatory variables. For example, RTs can be estimated to be shorter or longer in terms of their central tendency, but also narrower (less variable) or wider (more variable), depending on a given predictor (Bürkner, 2018; Kneib & Umlauf, 2017). As a result, distributional models provide better estimates of differences between means (because they relax the common assumption of equal variances), but more importantly, they allow going beyond the mean in order to draw inferences about how the whole shape of a response distribution is affected by the experimental manipulations (see Balota, Yap, Cortese, & Watson, 2008; Balota & Yap, 2011).^1^

**Fig. 1:**
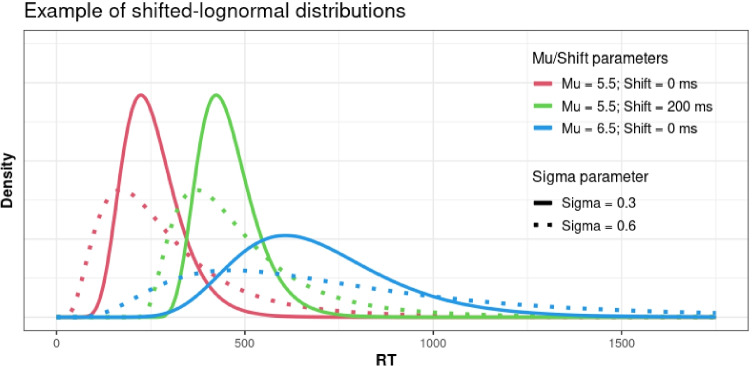
Examples of shifted-lognormal distributions, with varying *mu* (location), *sigma* (scale), and shift parameters. The *mu* and *sigma* parameters are expressed in log-milliseconds, whereas the shift is expressed in milliseconds. In a shifted-lognormal distribution, *mu* and *sigma* are the mean and standard deviation of the normal distribution of log(RT-shift). Conversely, an interpretable back-transformation of mu to the millisecond scale is given by exp(*mu*)+shift, which is equal to the median of RTs

Another advantage of Bayesian models is that they allow fitting virtually any kind of distribution in a straightforward way (Nicenboim, Logačev, Gattei, & Vasishth, 2016). Here, we modeled RTs as a ‘shifted-lognormal’ distribution, which is described by three parameters (see Fig. 1 for examples): (a) *mu*, the mean of (normally distributed) RTs in the log scale, (b) *sigma*, the standard deviation of (normally distributed) RTs in the log scale, and (c) *shift*, which moves the whole RT distribution to the right. The mean *mu* is a ‘location’ parameter, expressing central tendency, and can be taken as an index of difficulty (Wagenmakers & Brown, 2007): experimental manipulations that slow down responses, for example, can be empirically described as increasing *mu*, which disperses the RT distribution in the direction of longer reaction times. The standard deviation *sigma* is a ‘scale’ parameter, which stretches or squeezes the RT distribution around the same center. In this way, the *sigma* parameter captures variability of responses, and importantly, does so independently of their central tendency, that is, over and above the effects of condition difficulty (Wagenmakers & Brown, 2007). Finally, the shift is an estimated quantity in milliseconds that is added to the RT distribution and constitutes a lower bound for responses; this parameter may correspond to more peripheral (visual or motor) aspects of processing (Logan, 1992; Rouder, 2005).^2^ One advantage of including a shift parameter (relatively to assuming a lognormal distribution or to an analysis of log-RTs) is that shifted-lognormal models can more closely approximate the shape of RT distributions, and thus better satisfy the assumptions of linear models.

We made use of such distributional models to assess priming effects on both the *mu* and *sigma* parameters of the shifted-lognormal distribution of lexical decision RTs. In line with some of the previous morphological priming studies with L2 groups (Jacob et al., 2018; Kirkici & Clahsen, 2013; Veríssimo et al., 2018), we expected a difference between derivational and inflectional priming on the *mu* (mean) of log-RTs. Specifically, derived forms should produce facilitation effects on the recognition of their constituent stems, but the masked priming effects elicited by inflected forms may be smaller in magnitude or absent. Additionally, if L2 speakers show particular difficulties with consistently accessing and decomposing inflected forms in written word recognition, then the presentation of inflected primes may produce more inconsistent benefits, possibly leading to more variable lexical decision responses. In that case, inflected primes are expected to increase the *sigma* (standard deviation) of log-RTs in comparison to derived (and possibly unrelated) primes.

## Method

### Participants

Sixty-nine intermediate to advanced non-native speakers of English (54 women; 15 men) took part in the experiment in exchange for payment or course credits. Their mean age was 26.09 years (SD = 5.27, range = 18–37). All participants started learning English after the age of 4 (mean age of acquisition = 8.71, SD = 1.95, range = 4–13; 3 NAs). Participants were all native speakers of German, three of whom additionally spoke Russian as a native language. They all lived in Germany at the time of testing. All participants reported reading in English to some extent in their daily lives (mean use of English, as compared to other languages = 32.14%, SD = 18.10, range = 2–80%). Skill in English was tested by means of a 50-item multiple-choice grammar test adapted from the Oxford Placement Test 1 (Allan, 2004). Participants’ mean score was 38.62/50 (SD = 5.91; range = 21–48). This corresponds to a proficiency level roughly ranging between B2 and C2 of the Common European Framework of Reference for Languages (Council of Europe, 2001), i.e., from upper intermediate to highly proficient. All participants additionally took the English version of the LexTALE test (Lemhöfer & Broersma, 2012), a standardized vocabulary test consisting of an un-speeded visual lexical decision task. The LexTALE score is a percentage score, calculated as the percentage of correct responses corrected for the proportion of existing and non-existing words in the test. The group achieved a mean score of 78.91% (SD = 9.28; range = 61.25–98.75%, again roughly corresponding to B2 to C2 level). Prior to testing, all participants signed a written consent.

### Materials

The experiment included 102 English monomorphemic verbs used as targets (e.g., *print*). These were preceded by their *-ed* past-tense form (e.g., *printed*) as the inflected prime, their *-er* nominalization (e.g., *printer*) as the derived prime, or by an unrelated prime. Unrelated primes were dissimilar in form and meaning from their corresponding targets; half of them were *-ed* inflected forms and half of them were *-er* derived words. The distributions of word-form frequency, lemma frequency, and length (in letters) of the three prime types (inflected, derived, unrelated) were kept as similar as possible. Word-form and lemma frequency^3^ were extracted from the SUBTLEX-UK database (van Heuven, Mandera, Keuleers, & Brysbaert, 2014) and are provided in the Zipf scale, which approximately spans from 1 to 7; values below 3 indicate relatively low frequency, while values above 4 indicate high frequency. Item characteristics are provided in Table 1.

**Table 1:** Summary of the item characteristics (mean, SD, range)

Item type	Length (letters)	Word-form frequency	Lemma frequency
Target	5.09 (1.24)	3.93 (0.62)	4.04 (0.53)
	3–8	1.9–5.37	2.55–5.21
Derived prime	6.73 (1.07)	3.03 (0.64)	3.2 (0.65)
	4–9	1.3–4.22	1.6–4.46
Inflected prime	6.73 (1.07)	3.29 (0.73)	3.88 (0.58)
	4–9	1.17–4.76	2.23–5.11
Unrelated prime	6.73 (1.07)	3.24 (0.73)	3.66 (0.84)
	4–9	1.3–4.73	1.3–5.57

The 102 experimental targets and their corresponding inflected, derived, and unrelated primes were distributed across three presentation lists, following a Latin-Square design, so that each participant saw 34 targets associated with each of the three prime types. Three additional lists were created by reversing the order of the items in each list, for a total of six presentation lists. The 102 experimental prime-target pairs were mixed with 438 prime-target filler pairs, for a total of 540 items in each list. In all prime-target filler pairs, the prime was an existing word. Of the filler targets, 270 were non-existing words, generated from existing English words using the software Wuggy (Keuleers & Brysbaert, 2010). A ‘no’ response was therefore required in 50% of the 540 trials. Furthermore, 102 of the filler pairs included an inflected *-ed* prime (51) or a derived *-er* prime (51) combined with a non-existing word target (e.g., *barked-LEAMS*). This way, the presentation of *-ed* and *-er* primes did not represent a cue for lexicality of the target. Finally, 68 of the filler targets were non-existing words that were orthographically embedded in their primes (e.g., *sincere-SINCH*), so that form overlap was also not a cue for a ‘yes’ response. Overall, 25.2% of the prime-target pairs in each presentation list were related, either morphologically or orthographically.

### Procedure

Participants were tested in a quiet laboratory room. They were homogeneously assigned to one of the six lists. Participants’ accuracy and RTs in milliseconds were measured using the experimental software DMDX (Forster & Forster, 2003). We informed the participants that they would see a series of existing English words and invented words, and that they would have to indicate as quickly and as accurately as possible whether the word on screen is an existing word (‘lexical decision’). The ‘yes’ or ‘no’ responses were provided by pressing one of two buttons on a gamepad. Participants provided ‘yes’ responses with their dominant hand. Each trial started with a blank screen presented for 500 ms, followed by a mask consisting of ten hashes, also presented for 500 ms. Next, the prime appeared. The prime remained on screen for 50 ms and it was directly followed by the target. The target was displayed until a button press or until the timeout, which was set at 2000 ms. The next trial automatically began right after a button press or timeout. Primes were always presented in lowercase letters, and targets in uppercase letters.

### Data analysis

Incorrect responses (7.47%) and timeouts (0.18%) were excluded from analysis. There were no other exclusions of participants, items, or data points. No short outliers were identified (the fastest response was 309 ms). RTs were analyzed with Bayesian mixed-effects distributional regression models, assuming a shifted-lognormal response distribution. Bayesian models combine prior information with the evidence from the data in order to obtain a probability distribution over a parameter’s possible values—its *posterior distribution*. In this way, an experimental effect can be quantified in terms of the probability of its different magnitudes, which is more informative than a binary statement about whether an effect exists or not (McElreath, 2020; Vasishth, Nicenboim, Beckman, Li, & Kong, 2018). Prior distributions on all effects were centered around zero, with their width informed by domain knowledge of typical effect sizes in masked morphological priming (in L1 and L2). The chosen prior distributions ruled out effects that are extreme or unreasonable, but still allowed for a range of possible effects, both positive and negative (see Supplementary Materials S1, Table S1, for the full specification of prior distributions). Analyses were performed with the *brms* package in R (Bürkner, 2017; R Core Team, 2020). The procedures for fitting Bayesian models and assessing their convergence followed recent recommendations (Schad, Betancourt, & Vasishth, 2020; Vasishth et al., 2018).

The statistical models included fixed effects for prime type (unrelated, inflected, derived) and trial position (centered). The prime type variable was coded with treatment contrasts, with the unrelated condition as the reference value. Thus, the models directly estimated the effects of inflectional and derivational priming, more specifically, the differences between responses in the inflected and derived conditions relative to the unrelated condition. The fixed effects of prime type and trial were estimated on both the *mu* and *sigma* parameters of the shifted-lognormal RT distribution (i.e., on the mean and standard deviation of RTs in the shifted log scale). Models included random intercepts for participant and target word (for both *mu* and *sigma*). Random slopes for prime type were not included, because model comparisons on the basis of ELPD (a Bayesian measure of predictive accuracy) showed that none of the random slopes (by participant and by item, for mean and for *sigma*) provided meaningful improvements in predictive accuracy, defined here as an ELPD difference larger than 2 standard errors (Bürkner, 2017; Vasishth et al., 2018). All models and code can be downloaded from https://osf.io/4zwty.

## Results

Accuracy rates were very high in all conditions (unrelated: 90.15%; inflected: 93.73%; derived 93.69%). No further analyses of accuracy were conducted. Overall median RTs in the three prime type conditions were 661 ms (unrelated), 625 ms (inflected), and 625 ms (derived).

A summary of the statistical model is shown in Table 2. For each estimate in Table 2, we report the mean of its posterior distribution together with its 95% credible interval (i.e., the range within which a parameter falls with 95% probability). With regards to the *mu* parameter (see top four rows in Table 2), both morphological primes were found to speed up responses (see negative coefficients). That is, mean RTs (in the shifted log scale) were shorter following inflected and derived primes than following unrelated primes (by 43 ms for inflected, and 39 ms for derived, in back-transformed estimates). These effects show relatively narrow 95% credible intervals, with upper bounds that are far away from zero, and thus indicate strong support for facilitation effects on the speed of lexical decision responses for both types of morphological primes. The effects of inflected and derived primes can also be directly compared; in a Bayesian framework, this is easily achieved by subtracting all pairs of posterior samples for one and the other effect. The resulting posterior distribution shows little evidence for a difference between the effects of the two prime types on *mu* (mean -0.011 95%CI [-0.031, 0.008]).

**Table 2:** Summary of a distributional model, with both mean and standard deviation (sigma) of (shifted) log-RTs predicted by prime type (unrelated, inflected, derived)

	Estimate	L-95% CI	U-95% CI
Intercept (unrelated)	5.937	5.878	5.998
Prime type (derived vs. unrelated)	−0.110	−0.128	−0.091
Prime type (inflected vs. unrelated)	−0.121	−0.140	−0.102
Trial (centered)	−0.020	−0.025	−0.015
Sigma $\sim$ Intercept (unrelated)	0.314	0.297	0.332
Sigma $\sim$ Prime type (derived vs. unrelated)	0.010	−0.004	0.024
Sigma $\sim$ Prime type (inflected vs. unrelated)	0.025	0.010	0.040
Sigma $\sim$ Trial (centered)	0.001	−0.002	0.005

All estimates are in the shifted log-ms scale. The overall shift was estimated as 303 ms 95%CI: [298, 306]

The model also revealed effects of morphological primes on the *sigma* parameter, that is, on the standard deviation of RTs (again in the shifted log scale; see four bottom rows in Table 2). Specifically, the positive coefficients for both morphological priming conditions suggest an increase in variability relative to the unrelated condition.

**Fig. 2:**
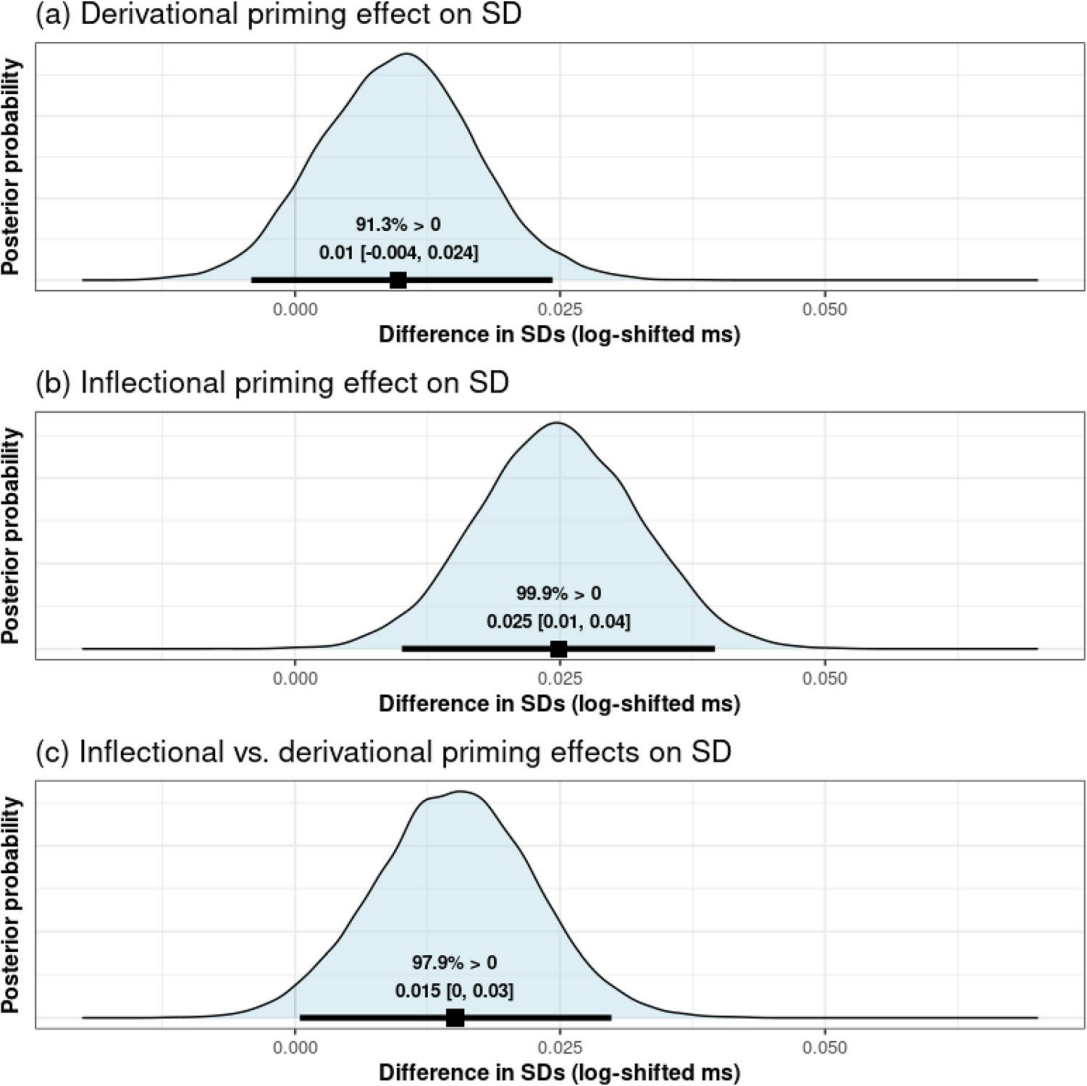
Posterior distributions for the effects of derived (panel a) and inflected primes (panel b) on the SDs of log-RTs, relative to unrelated primes, and for the comparison between inflected and derived primes (panel c). Each posterior distribution contains its mean and 95% credible interval displayed in numerical and graphical form, as well as the percentage of posterior samples that are on the positive side

Figure 2, panels a and b, show the full posterior distributions for these effects. For derived primes (panel a), a large proportion of the posterior distribution is on the positive side (i.e., greater standard deviation in the derived than in the unrelated condition). However, there is still some mass on the negative side, and the 95% credible interval for this effect crosses zero, indicating that very small or even negative values are not completely implausible (Lindley, 1970; Rouder, Haaf, & Vandekerckhove, 2018). There is much clearer support for an effect of inflected primes (again, relative to the unrelated condition), with almost all of its posterior distribution on the positive side (panel b). A visualization of the effects of prime type on *mu* and *sigma*, which shows the estimated RT distributions in the different prime type conditions, is included in the Supplementary Materials S2 (Figure S1).

Critically, the effect of inflected and derived primes can also be directly compared; as above, we obtained a posterior distribution for the contrast between the two effects, which is shown in Fig. 2, panel c. Although the difference between the two conditions is relatively small in magnitude, the exclusively positive 95% credible interval provides some evidence that inflected primes increased the standard deviation of (shifted log) RTs more than derived primes.^4^

## Discussion

Despite recommendations of looking beyond central tendencies of distributions in RT analyses (e.g., Heathcote, Popiel, & Mewhort, 1991), this rationale has hardly been applied to visual word recognition studies and it is virtually absent from morphological processing research (but see Balota et al., 2008; Hasenäcker, Beyersmann, & Schroeder, 2016; Yap, Balota, Cortese, & Watson, 2006). In the present study, we tried to close this gap and investigated masked morphological priming effects both on mean RTs to target words and on RT variability.

Based on the previous masked priming literature involving L2 speakers (Jacob et al., 2018; Kirkici & Clahsen, 2013; Silva & Clahsen, 2008; Veríssimo et al., 2018), we predicted priming effects on *mean* lexical decision RTs from derived words, but reduced or absent priming from inflected forms. However, our results showed that both types of morphologically related primes sped up word recognition latencies (and to a similar extent). Although unexpected, the finding of a priming effect with inflected words in L2 speakers has been previously reported in at least two other masked priming studies (Feldman et al., 2010; Foote, 2017), though these did not include derived primes. Our key prediction concerned effects of morphologically related primes on the standard deviation (*sigma*) of log-RTs, which was our measure of variability in responses. Following the proposal that variability in RTs should reflect the ability to efficiently access word representations (e.g., Segalowitz, 2008), and based on the previous evidence suggesting persistent vulnerabilities in L2 speakers in reliably accessing inflected forms (Blom et al., 2006; Hopp, 2013; White, 2003), we predicted that the presentation of inflected primes would produce more inconsistent benefits as compared to derived primes, leading to more variable lexical decision times. We found that the presentation of both derived and inflected primes led to larger RT variability as compared to unrelated primes, but inflected primes indeed increased RT variability more so than derived primes.

Our results suggest that, at least in an L2, accessing ‘print’ given ‘printed’ works less automatically than accessing ‘print’ given ‘printer’. Note that, although reliance on orthographic cues possibly plays a prominent role in L2 morphological processing (e.g., Heyer & Clahsen, 2015; Li, Taft, & Xu, 2017), this cannot explain our effect on RT variability, since the two morphological conditions were perfectly pairwise matched with regard to prime–target orthographic overlap. Therefore, the effect we report is more likely to result from specific differences between inflection and derivation. In spite of their superficial similarity, derived and inflected words modify their stems in different ways: while derivational operations create new lexical entries, inflectional operations are purely grammatical, in that they spell out morphosyntactic features of the stem, such as number, case, or tense (see Anderson, 1992). As mentioned in the Introduction, Segalowitz and Segalowitz (1993) and Solovyeva and DeKeyser (2018) have observed that variability increases in the process of establishing lexical representations, while it decreases when these are well established. We therefore suggest that the different lexical status of inflected and derived words is responsible for the differences in processing variability we observed: processing complex forms which have established lexical representations (in our case, derived words) and accessing the representations of their stems works more automatically, or efficiently, than processing complex forms that are bare spell-outs of grammatical properties of their stems (inflected forms). This should be particularly true in L2 populations, given that morphosyntax remains a particularly vulnerable domain in L2 acquisition and even proficient L2 speakers may not be able to consistently access the information contained in inflected forms (Blom et al., 2006; Hopp, 2013; White, 2003). In this way, the framework proposed by Segalowitz and Segalowitz (1993) and Solovyeva and DeKeyser (2018) can very well—and quite economically—account for our findings.

An open question is to what extent what we found also applies to native processing. Theoretically, the different nature of derived and inflected words may also affect the variability of responses in native speakers. Alternatively, the effects we have obtained may be specific to non-native or less proficient speakers, given their particularly vulnerability in the domains of inflection and morphosyntax. Future studies should examine morphological priming effects on RT variability across different populations, including native speakers and speakers at different proficiency levels. The detection of such effects in native speakers may nevertheless prove to be particularly challenging, considering that native speakers display higher language proficiency, less variable RTs, and show consistent and robust priming effects across all types of morphologically related primes, at least in the masked priming paradigm.

Accounts of morphological processing based on discriminative learning, which do not posit an independent level of morphological representation (e.g. Baayen, Chuang, & Blevins, 2018; Baayen et al., 2011; Baayen & Smolka, 2020), are also compatible with our data. A key tenet of discriminative learning is that words with similar meanings are more difficult to discriminate from each other. Consequently, words of an inflectional paradigm should be more difficult to discriminate than derived words, as the former are characterized by high semantic similarity between each other. Accessing the meaning of inflected words should therefore lead to greater inconsistencies as compared to derived words, resulting in larger RT variability. This problem is likely to be particularly strong for L2 speakers, since they are faced with the near-synonymy of translation equivalents between their L1 and their L2 (see Chuang, Bell, Banke, & Baayen, 2021a). However, considering the small size of English inflectional paradigms, it would remain to be tested whether English inflected words are indeed more difficult to discriminate than English derived words, especially in the case of the very productive and transparent *-er* derivations that were tested in the present study.

A relevant aspect of the study concerns the type of RT distribution that we modelled. Similarly to the studies on RT variability reported above, our goal was to investigate variability in responses as measured separately from their central tendency. However, in RT distributions, the mean and standard deviation of the distribution have a linear relationship (Wagenmakers & Brown, 2007), such that manipulations that slow down responses (i.e., lead to larger mean RTs in milliseconds) also increase their variability (i.e., lead to larger SDs). By modeling a (shifted) lognormal distribution, we could estimate effects on the two parameters of interest, *mu* and *sigma*, independently of one another, thus being able to isolate effects on variability from bare speed-up effects. This is very well illustrated by the fact that derived and inflected primes had different effects on *sigma* (i.e., on the standard deviation of shifted log-RTs), although the two prime types were associated with very similar effects on *mu* (i.e., on the mean). Note, however, that the shifted-lognormal distribution is only one of the possible distributions that can be used to model RT data. Other theoretical distributions that can capture the properties of RT data are the gamma and the inverse-Gaussian distributions (see Lo & Andrews, 2015). However, these are characterized by different parameters (e.g., the tail *tau*) and different relationships between mean and SD. Therefore, it is worth stressing that the effects we are reporting here pertain to the SD of a distribution of shifted log-RTs, and may not apply to other distributions.

To conclude, we have shown that morphological type (derivation vs. inflection) modulates the processing of complex words as tested with the masked priming paradigm, with inflected primes in particular producing larger increases in the variability of responses. By examining only the central tendency (i.e., the mean) of response times, we would have been unable to distinguish priming effects produced by derived and inflected words. However, the differences between the two prime types emerged when going beyond mean RTs by specifically investigating the variability of responses. Besides the relevance of the present results for models of morphological processing, the current study therefore emphasizes the importance of testing predictions on other parameters of the RT distribution rather than just its central tendency (Balota et al., 2008; Heathcote et al., 1991), and more generally, of treating variability in performance as a direct object of investigation in psycholinguistics.

## Supplementary Information

Below is the link to the electronic supplementary material.

Supplementary file 1 (PDF 1.59 MB)

## Data Availability

$\bullet$ S1. Prior distributions on each estimate of the Bayesian distributional model $\bullet$ S2. Plot of estimated distributions in the different prime type conditions $\bullet$ S3. Alternative analysis with generalized additive mixed models (GAMMs)

## Footnotes

1 Effects on both the mean and the standard deviation of a dependent variable can also be tested with generalized additive mixed models (GAMMs; see Baayen, Vasishth, Kliegl, & Bates, 2017) with the *mgcv* package (Wood, 2017) in R. We therefore provide an alternative analysis of our data with GAMMs in the Supplementary Materials S3. We thank Harald Baayen for this useful suggestion.

2 We here refer to common interpretations of the different parameters of RT distributions. However, note that whether these parameters neatly map onto specific cognitive processes has also been a matter of debate (see Matzke & Wagenmakers, 2009).

3 Lemma frequency here refers the dominant word class of the word. This means that, for words that can belong to different word classes (as is often the case in English; see e.g., ‘brew$_\mathsf{noun}$’ and ‘brew$_\mathsf{verb}$’), the word class that is considered for lemma frequency is the one most frequently associated to that word, in the Subtlex-UK corpus.

4 We fitted additional models to address possible confounds of the priming effects on *sigma*. First, effects on *sigma* could arise from unmodelled between-participant variability (i.e., from the lack of random slopes) in priming effects on *mu*. Second, there are some differences between derived and inflected primes in their mean word form and lemma frequencies (see Table 1). However, models containing by-participant and by-item random slopes on *mu*, as well as models with frequency measures as covariates, revealed very similar effects to those reported in Fig. 2. We conclude that the reported priming effects are not explainable by between-subject heterogeneity or by frequency differences. We thank Seamus Donelly for these suggestions.
