# Supplementary material for: Investigating variability in morphological processing with Bayesian distributional models
Source: Psychon Bull Rev. 2022 Jun 17;29(6):2264–74. doi: 10.3758/s13423-022-02109-w (PMC9722799; doi:10.3758/s13423-022-02109-w)
Supplement: Supplementary file 1 — (PDF 1.59 MB) [file 13423_2022_2109_MOESM1_ESM.pdf]

Laura Anna Ciaccio and João Veríssimo

Supplementary Materials to:
Investigating variability in morphological processing with Bayesian distributional models

S1. Prior distributions on each estimate of the Bayesian distributional model

Table S1: Prior distributions on each estimate of the Bayesian distributional model

Parameter	Coefficient	Type	Prior	Units
Mu (mean)	Intercept	Fixed effect	Normal(6.0, 0.5)	log-ms
Mu (mean)	Intercept	Random effect	Normal(0, 0.5)	log-ms
Mu (mean)	Slopes	Fixed effect	Normal(0, 0.25)	log-ms
Sigma (SD)	Intercept	Fixed effect	Normal(-2, 1)	log of log-ms
Sigma (SD)	Intercept	Random effect	Normal(0, 1)	log of log-ms
Sigma (SD)	Slopes	Fixed effect	Normal(0, 0.5)	log of log-ms
Shift			Normal(250, 100)	ms

Note. Prior distributions are in the notation Normal(mean, SD). These are expressed in different units due to the parametrizations used by the brms R-package: estimates on mu (mean) are in log-ms because a (shifted-)lognormal response distribution was assumed; estimates on sigma (SD) are modelled in the log-scale by default because they must be strictly positive (hence, in log of log-ms). Note that all sigma effects reported in the paper were back-transformed to log-ms, for easier interpretation and for consistency with the other estimates.

S2. Plot of estimated distributions in the different prime type conditions

Figure S1 below shows the estimated RT distributions in the three prime type conditions (unrelated, inflected, derived), with estimates obtained from the Bayesian distributional model reported in the main paper. RTs are shown in the modelled shifted log-millisecond scale; thus, they are normally distributed with estimated *mu* and *sigma* parameters. Plotting in a log scale allows bypassing the dependency between mean and standard deviation that exists for millisecond RTs, so that the effects of prime type can be visualised independently.

Note that, in a Bayesian framework, every quantity (i.e., every estimated parameter and every prediction) is associated with a full posterior distribution. Visualisation of distributions can thus become quite complex, as well as computationally intensive. The visualisation below is a simplification: it does not show the uncertainty associated with the predictions, but depicts only the idealised normal distributions that were estimated by the model, on the basis of point estimates (i.e., the mean of the posteriors) of both *mu* and *sigma*, for each of the three conditions.

As can be seen in Fig. S1, prime type had effects on both *mu* and *sigma*. The model estimated shorter mean RTs in the inflected and derived conditions than in the unrelated condition. With regards to *sigma*, the effects were small, but can be seen by inspecting the heights of the three distributions: responses in the derived condition were estimated as slightly more variable than in the unrelated condition ('shorter' distribution), and those in the inflected condition were more variable than both.

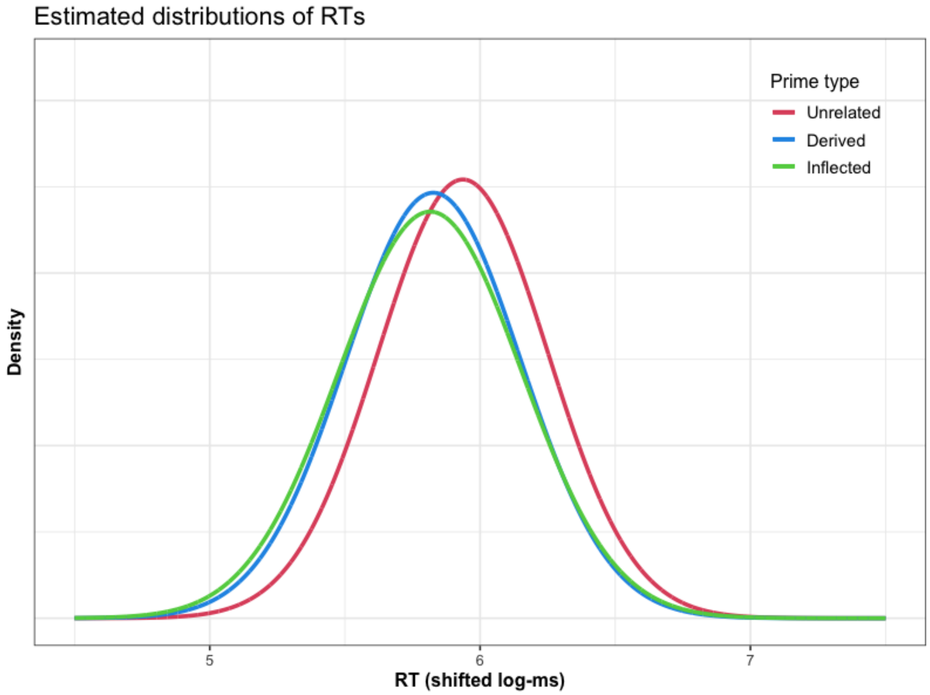

Figure S1: Estimated distributions of RTs in the three prime type conditions (unrelated, inflected, derived). RTs are shown in the shifted log millisecond scale.

S3. Alternative analyses with Generalized Additive Mixed Models (GAMMs)

The present supplementary materials illustrate an additional way of analyzing reaction time (RT) distributions by making use of Generalized Additive Mixed Models (GAMMs); see Baayen et al. (2017)[1]. Similarly to the Bayesian distributional model reported in the main paper (Ciaccio & Veríssimo, 2022), GAMMs allow to test for experimental effects on both the mean of (transformed) RTs, i.e. the *mu* parameter of the distribution, and their standard deviation (SD), i.e. the *sigma* parameter. This can be achieved by specifying `family = gaulss`. This way, we fit a Gaussian model containing two formulae, the first specifying the predictors for estimating effects on mean RTs, and the second the predictors for effects on their SD.

All analyses were performed using the `mgcv` package (Wood, 2017) version 1.8.40 in R version 4.2.0 (2022-04-22) (R Core Team, 2020). All models and code can be downloaded from https://osf.io/4zwty.

We first load the data and prepare the dataset for the analyses.

```
c. <- function(x) scale(as.numeric(x, scale=F)) # Function used to center variables

# Load data
varmorph <- read.csv("data/varmorph.csv", sep=",", header=T) # Read data

varmorph$set <-  as.factor(varmorph$set)
varmorph$subj_id <-  as.factor(varmorph$subj_id)
varmorph$target <- as.factor(varmorph$target)
varmorph$prime_type <-  as.factor(varmorph$prime_type)

c_trialN <- c.(I(varmorph$trial_n/100)) # Center trial number

# - Keep only experimental items (exclude fillers)
# - Exclude incorrect responses and timeouts
# - Set unrelated prime as baseline

varmorph <- droplevels(varmorph[varmorph$set=="experimental",])
varmorph <- droplevels(varmorph[varmorph$accuracy==1,])
varmorph$prime_type <-  relevel(varmorph$prime_type, "unrelated")
```

[1] We thank Harald Baayen for suggesting this to us and providing useful instructions.

Before fitting our Gaussian model, we need to transform our RT data with an appropriate transformation. The transformation that best corresponds to the shifted log-normal distribution that we fitted in the main paper consists in log-transforming RTs after subtracting the 'shift', i.e. after shifting the entire distribution by an amount of milliseconds under which RTs are considered implausible. In our case, the 'amount of ms' we take is the shift estimated by the Bayesian distributional model reported in the main paper, i.e. 302.58 ms. As we can see from the plots below, shifted log-transforming our data (left panel) makes them closer to a Gaussian distribution than a simple log-transformation (middle panel).

Admittedly, taking a shift estimated by another Bayesian distributional model may make the analysis unnecessarily complex. At least for this dataset, the inverse transformation seems to also work quite well (see right panel below). For those analyzing data directly with GAMMs, this may be a more practical option, though probably at the expense of interpretability of the estimates. In this document, we will stick to the shifted log transformation for consistency with the main paper.

```
# log transformation
varmorph$RTlog <-  log(varmorph$rt)

# shifted log-transformation, with estimated shift from Bayesian model (see main paper)
varmorph$RTslog <- log(varmorph$rt-302.58)

# inverse transformation
varmorph$RTinv <- -1000/varmorph$rt

par(mfrow=c(1,3))


qqnorm(varmorph$RTlog) # log transformation does not work so well (left panel)
qqline(varmorph$RTlog)

qqnorm(varmorph$RTslog) # shifted log transformation is better (middle panel)
qqline(varmorph$RTslog)

qqnorm(varmorph$RTinv) # inverse reaction times are also fine (right panel)
qqline(varmorph$RTinv)
```

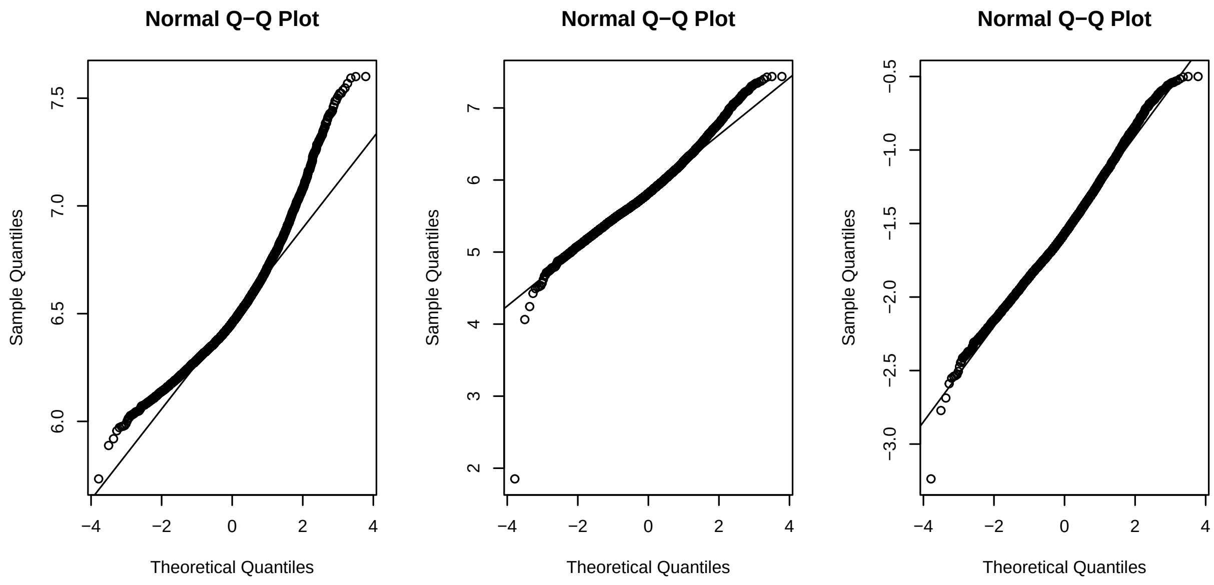

In the following, we will start from a simple GAMM and make it successively more complex, leading to a final model with a very similar structure to the one reported in the main manuscript. We will additionally show that: (a) the final model testing effects on both the *mu* and the *sigma* parameters has a better fit than one only testing effects on *mu*; (b) the final model shows parallel results to the one reported in the manuscript regarding the main contrast we focused on, namely the difference between inflection and derivation with regards to the effect on *sigma*.

We first fit a baseline mixed-effects model (`m0`) testing only effects of Prime Type (inflected, derived, unrelated) on *mu*, i.e. on mean (shifted log) reaction times. The model contains by-subject and by-item random effects. It additionally includes the predictor (centered) Trial Number. Unlike in the main paper, because GAMMs allow for smooth predictors and the effect of trial number over the experiment is likely to be non-linear, we specify Trial Number as a smooth term by using `s()`. Starting from this model, we will now fit more complex models and evaluate whether they provide a better fit than the simpler model based on their `REML` score (lower `REML` = better fit).

```
m0 <-  gam(RTslog ~ prime_type + s(c_trialN) +
             s(subj_id, bs="re") +
             s(target, bs="re"),
           data=varmorph, method="REML")
```

```
m0$gcv.ubre[1] # Get the REML
```

```
##     REML
## 2432.698
```

We next add the `sigma` parameter to our model (`m1`), and additionally add (centered) Trial Number as smooth predictor to the sigma part of the formula (`m2`). Note that, for consistency with the model reported in the main paper, we will keep `m2` although Trial Number does not improve the fit of `m1`. Therefore, at this step, the model fit after adding `sigma` is slightly worse than the fit of `m0`. However, we haven't included random effects for the `sigma` parameter yet.

```
m1 <-  gam(list(RTslog ~ prime_type + s(c_trialN) +
                  s(subj_id, bs="re") +
                  s(target, bs="re"),
                ~ prime_type),
           data=varmorph, family="gaulss", method="REML")

m2 <-  gam(list(RTslog ~ prime_type + s(c_trialN) +
                  s(subj_id, bs="re") +
                  s(target, bs="re"),
                ~ prime_type + s(c_trialN)),
           data=varmorph, family="gaulss", method="REML")
```

```
# Get the REML of m1 (left) and m2 (right)
c(m1$gcv.ubre[1],m2$gcv.ubre[1])
```

```
##     REML     REML
## 2431.954 2433.578
```

Adding by-item random effects (m3) improves model fit compared to all previous models, including those not containing the sigma parameter. Therefore, the presence of the sigma parameter seems to be justified.

```
m3 <-  gam(list(RTslog ~ prime_type + s(c_trialN) +
                 s(subj_id, bs="re") +
                 s(target, bs="re"),
               ~ prime_type + s(c_trialN) +
                 s(target, bs="re")),
           data=varmorph, family="gaulss", method="REML")
```

```
m3$gcv.ubre[1] #  lower REML than m2 and m0
```

```
##     REML
## 2391.449
```

Adding by-subject random effects (m4) further improves the model fit. Adding by-subject or by-item random slopes did not further improve the model fit (models including random slopes can be downloaded at https://osf.io/4zwty). Therefore, m4 is our final model. This has a similar structure to the one reported in the main manuscript. It provides a better fit than a model which only tests effects on mu (cfr. REML m4 = 2294.1, see summary below; vs. REML m0 = 2432.698). The residuals also seem fairly normal.

```
m4 <-  gam(list(RTslog ~ prime_type + s(c_trialN) +
                 s(subj_id, bs="re") +
                 s(target, bs="re"),
               ~ prime_type + s(c_trialN) +
                 s(subj_id, bs="re")+
                 s(target, bs="re")),
           data=varmorph, family="gaulss", method="REML")
```

```
qqnorm(resid(m4))
qqline(resid(m4))
```

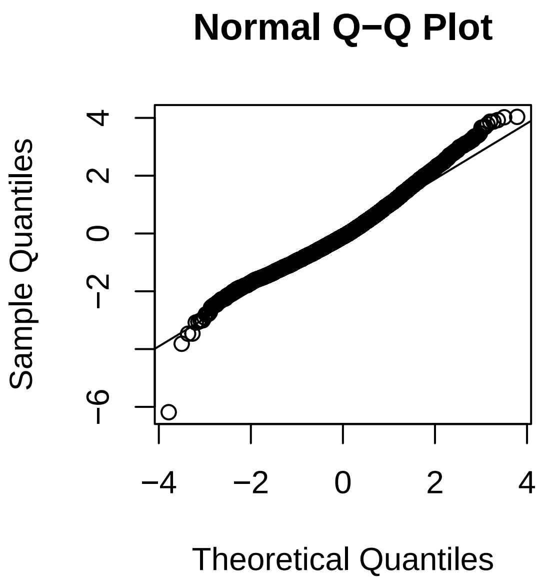

Printing the summary of the model, we can now evaluate its output. Note that, for the *mu* parameter, model estimates are provided in the same scale as the data to which we fitted the model (i.e., in the log scale). Instead, for the *sigma* parameter, they are in the log of log-ms. Concerning the effects of 'Prime Type' on *mu*, the model output shows similarly large priming effects (i.e., speed-ups in RTs) on mean RTs for inflected and derived primes (respectively, β= -0.1212462 and β=-0.1098143). Regarding effects on *sigma*, while both inflected and derived primes *increase* RT variability, this effect seems to be robust only for inflected primes, and it is larger for inflected primes than for derived primes (respectively, β=0.0810701 and β=0.0326677).

```
summary(m4)
```

```
##
## Family: gaulss
## Link function: identity logb
##
## Formula:
## RTslog ~ prime_type + s(c_trialN) + s(subj_id, bs = "re") + s(target,
##     bs = "re")
## ~prime_type + s(c_trialN) + s(subj_id, bs = "re") + s(target,
##     bs = "re")
##
## Parametric coefficients:
##                        Estimate Std. Error z value Pr(>|z|)
## (Intercept)            5.935188   0.028898 205.385  < 2e-16 ***
## prime_typederived     -0.109814   0.009369 -11.720  < 2e-16 ***
## prime_typeinflected   -0.121246   0.009592 -12.640  < 2e-16 ***
## (Intercept).1         -1.215314   0.028443 -42.728  < 2e-16 ***
## prime_typederived.1    0.032668   0.023323   1.401 0.161313
## prime_typeinflected.1  0.081070   0.023347   3.472 0.000516 ***
## ---
## Signif. codes:  0 '***' 0.001 '**' 0.01 '*' 0.05 '.' 0.1 ' ' 1
##
## Approximate significance of smooth terms:
##                   edf  Ref.df   Chi.sq p-value
## s(c_trialN)     1.001   1.001   61.609  <2e-16 ***
## s(subj_id)     66.228  68.000 2997.768  <2e-16 ***
## s(target)      92.963 101.000 1495.619  <2e-16 ***
## s.1(c_trialN)   1.002   1.002    0.546   0.461
## s.1(subj_id)   55.626  68.000  468.930  <2e-16 ***
## s.1(target)    62.877 101.000  296.472  <2e-16 ***
## ---
## Signif. codes:  0 '***' 0.001 '**' 0.01 '*' 0.05 '.' 0.1 ' ' 1
##
## Deviance explained = 40.3%
## -REML = 2294.1  Scale est. = 1         n = 6512
```

Finally, by releveling the baseline to `derived`, we can extract the model estimates for the direct comparison between derived and inflected Prime Type. Inflected and derived primes reliably differ for their effect on *sigma*, with inflected primes increasing RT variability more than derived primes, as reported in the main paper.

```
varmorph$prime_type <-  relevel(varmorph$prime_type, "derived")
m4der <- update(m4)
```

```
summary(m4der)$p.table
```

```
##                           Estimate  Std. Error    z value     Pr(>|z|)
## (Intercept)             5.82537407 0.028908640 201.509796 0.000000e+00
## prime_typeunrelated     0.10981431 0.009369494  11.720410 1.001859e-31
## prime_typeinflected    -0.01143185 0.009621812  -1.188118 2.347870e-01
## (Intercept).1          -1.18264655 0.028222184 -41.904856 0.000000e+00
## prime_typeunrelated.1 -0.03266774 0.023322960  -1.400669 1.613131e-01
## prime_typeinflected.1  0.04840240 0.023063800   2.098631 3.584944e-02
```

References

Baayen, R. H., Vasishth, S., Kliegl, R., & Bates, D. (2017). The cave of shadows: Addressing the human factor with generalized additive mixed models. *Journal of Memory and Language*, *94*, 206–234. https://doi.org/10.1016/j.jml.2016.11.006

Ciaccio, L. A., & Veríssimo, J. (2022). Investigating variability in morphological processing with Bayesian distributional models. *Psychonomic Bulletin & Review*, *Advance online publication*. https://doi.org/10.3758/s13423-022-02109-w

R Core Team. (2020). *R: A language and environment for statistical computing*. R Foundation for Statistical Computing.

Wood, S. N. (2017). GAMs in Practice: mgcv. In *Generalized Additive Models* (2nd ed.). Chapman and Hall/CRC Press.
